# Predictive role of cardiopulmonary bypass exposure indexed to body surface area on postoperative organ dysfunction: a retrospective cohort study

**DOI:** 10.1093/icvts/ivae171

**Published:** 2024-10-07

**Authors:** Florian Falter, Ryan Salter, Jose Fernandes, Christiana Burt, Kate Drummond, Ganesh Ramalingam, Samer Nashef

**Affiliations:** Department of Anaesthesia and Intensive Care, Royal Papworth Hospital, Cambridge, UK; Department of Anaesthesia and Intensive Care, Wellington Hospital, Wellington, New Zealand; Department of Clinical Perfusion, Royal Papworth Hospital, Cambridge, UK; Department of Anaesthesia and Intensive Care, Royal Papworth Hospital, Cambridge, UK; Department of Anaesthesia, Royal Adelaide Hospital, Adelaide, Australia; Department of Anaesthesia and Intensive Care, Royal Papworth Hospital, Cambridge, UK; Department of Cardiothoracic Surgery, Royal Papworth Hospital, Cambridge, UK

**Keywords:** Cardiopulmonary bypass, Organ dysfunction, Acute kidney injury, Atrial fibrillation, Length of stay

## Abstract

**OBJECTIVES:**

Long cardiopulmonary bypass times are associated with adverse postoperative outcomes and increased healthcare resource use. It is likely that this effect is pronounced in smaller patients. Previous studies have been criticized for not taking into consideration that prolonged bypass times are often due to higher complexity. The purpose of this study was to investigate the relationship between bypass index (bypass time/body surface area) and adverse postoperative events.

**METHODS:**

Single-centre, retrospective cohort study including 2413 patients undergoing cardiac surgery on cardiopulmonary bypass from June 2018 to April 2020. Length of hospital stay, as surrogate marker of postoperative morbidity, was selected as primary outcome. The strength of association between bypass index and the primary outcome was assessed with linear regression analysis. Secondary outcomes included new onset renal, pulmonary or cardiac rhythm dysfunction. The predictive value of bypass index was assessed with linear regression analysis; univariate and multiple regression were used to assess the strength of association between Bi and the secondary outcomes.

**RESULTS:**

Bypass index was predictive for length of stay at univariate (Relative Risk (RR): 1.004, *P* < 0.001) and at multivariable (RR: 1.003, *P* < 0.001) analysis. The association between bypass index and new renal (mean difference: 14.1 min/m^2^, *P* < 0.001) and cardiac rhythm dysfunction (mean difference: 12.6 min/m^2^) was significant. This was not true of postoperative lung dysfunction (mean difference: −1.5 min/m^2^, *P* = 0.293).

**CONCLUSIONS:**

Bypass index, calculated as total bypass time/patient body surface area, is predictive of postoperative morbidity and resource utilization after cardiac surgery on pump.

## Graphical Abstract

**Figure:**
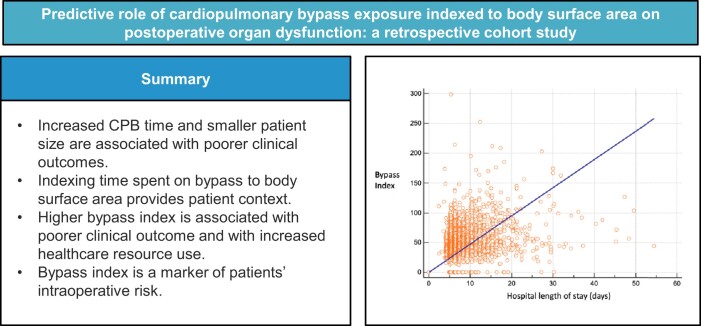

## INTRODUCTION

Since its inception in the 1950s, cardiopulmonary bypass (CPB) has been an essential component of cardiac surgery. Early experience demonstrated an association between CPB exposure and postoperative organ dysfunction, morbidity and mortality [1–3]. CPB has been shown to activate coagulation, fibrinolysis [4] and platelets [5]. Multiple inflammatory mediators have been implicated in contributing to organ dysfunction [6]; key drivers for the inflammatory response appear to be air-blood interface [7], circuit artificial surface [8] and re-infusion of shed pericardial blood [9].

Longer CPB time has been associated with increased duration of mechanical ventilation [10] and higher risk of surgical site infection [11], kidney injury [12], organ failure [13], and mortality [14]. It is conceivable that the risk of CPB-associated complications correlates inversely with patient size. Lower body surface area (BSA) is associated with increased haemodilution at CPB initiation [15] and increased odds of allogeneic red blood cell transfusion [16]. High body mass index (BMI) has been associated with lower odds of mortality in patients undergoing cardiac surgery [17].

The purpose of the current study is to investigate the role of a bypass index (BI), calculated as total CPB time divided by the patient BSA. To the best of our knowledge, no prior study has investigated the predictive role of bypass time indexed to patient size on postoperative morbidity and mortality, despite biological plausibility.

## METHODS

This study was designed utilizing the STROBE checklist for observational studies [18]. We conducted a single-centre, retrospective, cohort study of adult patients (18 years and over) undergoing elective or semi-urgent cardiac surgery on CPB at an academic, quaternary referral hospital between 1 June 2018 and 30 April 2020. Patients having deep hypothermic circulatory arrest, solid organ transplant, major aortic surgery, pulmonary thrombo-endarterectomy, urgent (<8 h) and emergent surgery (<4 h) were excluded as were those in end-stage renal failure requiring haemodialysis and those who died within the first 30 days after surgery. Anaesthesia and intensive care data were retrieved from Metavision (iMDsoft, Duesseldorf, Germany), and patient demographics data, risk and surgical data were retrieved from an in-house database, CARDS II.

The key exposure of interest was the BI. The primary outcome was the strength of association between BI and hospital length of stay (LOS) as a surrogate marker for morbidity (Table 1). Secondary outcomes included the strength of association between BI and new renal (creatinine rise >50% on postoperative day 1), pulmonary (PaO_2_/FiO_2_ <200 during the first 6 h postoperatively) and cardiac rhythm dysfunction (new-onset atrial fibrillation within 24 h postoperatively), intensive care unit (ICU) LOS. We included a pre-specified subgroup of patients undergoing isolated coronary artery bypass grafting (CABG) and undertook a post hoc analysis assessing the primary outcome with CPB time only as the exposure of interest to investigate whether BI provides additional value as compared to CPB time alone.

**Table 1:** Hospital length of stay (death within 30 days excluded)

	Univariate beta coefficient (95% CI)	*P*-value	Multivariable beta coefficient (95% CI)	*P*-value
BI (per unit)	0.004 (0.003, 0.005)	<0.001	0.034 (0.027, 0.094)	<0.001

CI: confidence interval; BI: bypass index.

### Anaesthesia and perfusion technique

Please see the Supplementary material.

### Statistical methods

Continuous variables are presented as mean ± standard deviation (SD) and median—interquartile range (IQR) for normally and non-normal distributed data, respectively. Categorical variables are reported as counts and percentages.

The strength of association between BI or CPB time and LOS was assessed using negative binomial regression analysis. Unadjusted comparison of means, univariate and multiple regression analyses were carried out to examine whether BI is associated with new postoperative cardiac rhythm disturbance, new renal dysfunction, new pulmonary dysfunction and 30-day mortality. Variables included in multivariable regression analyses were BI, sex, age and BMI. Where regression analyses were undertaken, binary outcome variables were reported using the odds ratio, and continuous outcome variables using the beta coefficient.

Following first analysis we investigated the association between ICU and hospital LOS and BI. Generalized additive model (GAM) framework was used to model potential non-linear relationships and interactions between predictors. GAMs were fitted using the geom_smooth() function in ggplot2 with method = ‘gam’, incorporating a smooth term for BI to capture non-linear effects of the continuous predictor BI, and categorical predictor of comorbidities representing clinical presentations of pulmonary, cardiac and renal dysfunction as well as combinations of them. Model parameters were automatically adjusted through penalized likelihood estimation, ensuring robustness in modelling complex relationships.

Secondary analyses were undertaken to further investigate any association between BI and postoperative organ dysfunction in patients undergoing CABG only.

Statistical analysis was performed using the Stata software package (StataCorp, College Station, TX, USA) and in the statistical programming environment R Version 4.4.1 (Vienna, Austria) using the mgcv package where necessary and ggplot2 for visualization of results.

## RESULTS

From 1 June 2018 to 30 April 2020, 2413 patients met the inclusion criteria. 119 patients were excluded due to missing data (14 due to missing BSA data, 105 due to missing outcome data). 2294 patients were included in our final analysis. Demographic is summarized in Table 2.

**Table 2:** Summary statistics

Male, *n* (%)	1687 (26.6%)
Female, *n* (%)	610 (26.5%)
Age, mean (SD)	68.8 (11.1)
Height (cm), mean (SD)	169.9 (9.5)
Weight (kg), mean (SD)	82.6 (16.8)
BSA, mean (SD)	1.97 (0.23)
EuroSCORE 2, mean (SD)	3.15 (4.35)
Preoperative rhythm	
Sinus, *n* (%)	1897/2271 (82.6%)
AF/flutter, *n* (%)	314/2271 (13.8%)
Other, *n* (%)	60/2271 (2.6%)
Type of surgery	
CABG, *n* (%)	840/2297 (36.6%)
Valve, *n* (%)	690/2297 (30.0%)
Double valve, *n* (%)	105/2297 (4.6%)
Triple valve, *n* (%)	11/2297 (0.5%)
CABG + valve, *n* (%)	404/2297 (17.6%)
Aortic, *n* (%)	167/2297 (7.3%)
Redo, *n* (%)	59/2297 (2.6%)
Other, *n* (%)	21/2297 (0.9%)
Total bypass time (min), mean (SD)	108.7 (57.5)
Bypass index, mean (SD)	55.9 (31.2)
Any organ dysfunction, *n* (%)	960/2297 (41.8%)
New postoperative rhythm dysfunction, *n* (%)	304/2288 (13.3%)
Postoperative renal dysfunction, *n* (%)	90/2276 (4%)
Postoperative CRRT, *n* (%)	31/2297 (1.3%)
Postoperative pulmonary dysfunction, *n* (%)	724/2293 (31.5%)

SD: standard deviation; BSA: body surface area; AF: atrial fibrillation; CABG: coronary artery bypass surgery; CRRT: continuous renal replacement therapy.

### Primary outcome

Primary outcome analyses demonstrated predictive utility of BI on hospital LOS at both univariate (beta coefficient: 0.004 days per BI unit, 95% CI: 0.003–0.005, *P* < 0.001) and multivariable (beta coefficient: 0.003 days per BI unit, 95% CI: 0.002–0.004, *P* < 0.001) levels.

Supplementary Material, Table S1 presents a comparison of the baseline and the postoperative variables between patients with hospital LOS < median and those with hospital LOS ≥ median. This comparison confirms that patient factors such as age as well as intraoperative factors such as CPB time and BI or postoperative factors such as organ dysfunction are all associated with increased LOS.

**Figure 1:**
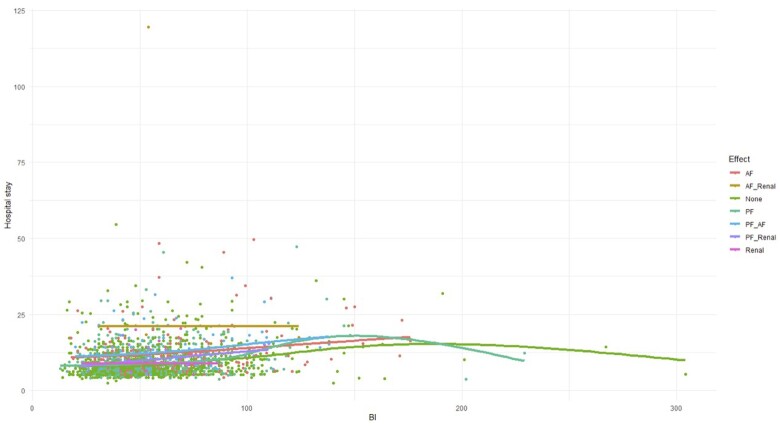
Scatter plot of BI vs. hospital length of stay using a generalized additive model. BI: bypass index.

Applying the GAM revealed a significant non-linear relationship between BI and hospital LOS (Fig. 1). Higher BI was associated with an increased LOS, with diminishing returns observed at higher BI.

### Secondary outcomes

At logistic regression analysis, BI was predictive of new renal dysfunction and new cardiac rhythm disturbance at univariate and multivariable regression analyses, but not pulmonary dysfunction. BI was associated with increasing ICU LOS at univariate (beta coefficient: 0.011 days per BI unit, *P* < 0.001) and multivariable analysis (beta coefficient 0.011 days per BI unit, *P* < 0.001) (Table 3).

**Table 3:** Univariate and multivariable regression analysis

			Univariate OR (95% CI)	*P*-value	Multivariable OR (95% CI)	*P*-value
Any postoperative organ dysfunction		BI^a^	1.005 (1.002, 1.009)	0.001	1.009 (1.005, 1.012)	<0.001
Postoperative cardiac dysfunction		BI^a^	1.013 (1.009, 1.017)	<0.001	1.014 (1.01, 1.019)	<0.001
Postoperative renal dysfunction		BI^a^	1.001 (1.001, 1012)	0.022	1.007 (1.001, 1.013)	0.013

			Univariate beta coefficient (95% CI)	*P*-value	Multivariable beta coefficient (95% CI)	*P*-value

ICU LOS		BI^a^	0.011 (0.01, 0.012)	<0.001	0.011 (0.01, 0.012)	<0.001

a BI per unit.

OR: odds ratio; CI: confidence interval; BI: bypass index; ICU: intensive care unit; LOS: length of stay.

**Figure 2:**
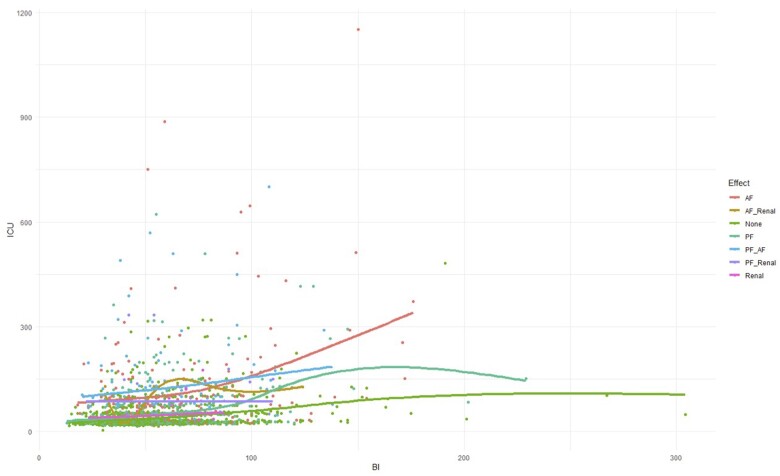
Scatter plot of BI vs. ICU length of stay using a generalized additive model. BI: bypass index; ICU: intensive care unit.

Applying GAMs showed a statistically significant non-linear relationship between BI and length of ICU stay. The most significant predictor of increased ICU LOS was cardiac dysfunction. The increase in stay continued to increase non-linearly as BI increased, without showing any diminishing returns (Fig. 2).

We undertook a post hoc analysis to see if BI was more predictive of outcome than CPB time alone. At regression analysis increasing CPB time was found to be predictive of increased hospital LOS in univariate and multivariable models. Furthermore, CPB time was significantly higher in patients who developed any new organ dysfunction, new cardiac rhythm disturbance, and new renal dysfunction (Table 4).

**Table 4:** Primary outcome for non-indexed CPB time

Hospital length of stay (death within 30 days excluded)				
	Univariate beta coefficient (95% CI)	*P*-value	Multivariable beta coefficient (95% CI)	*P*-value
CPB time (per minute)	0.002 (0.002, 0.003)	<0.001	0.019 (0.015, 0.024)	<0.001

CPB: cardiopulmonary bypass; CI: confidence interval.

## DISCUSSION

We retrospectively studied adult patients undergoing elective or semi-urgent cardiac surgery on CPB to investigate the utility of indexing bypass time to BSA, creating a BI. Our primary outcome was postoperative LOS and we found that higher BI was associated with increased postoperative LOS in hospital as well as in ICU. Further analyses demonstrated a positive association between increasing BI and risk of new renal dysfunction and new cardiac rhythm disturbance. On regression analyses, we found that BI possessed a similarly strong association as CPB time alone.

CPB flow is generally adjusted to BSA and proponents of more individualized approaches like goal-directed perfusion are hoping to ameliorate the negative effects of extracorporeal circulation in patients although results are contradictory [19, 20]. The negative effects of CPB, however, are dose—i.e. time—dependent. We, therefore, proposed the BI as a means of more accurately quantifying bypass exposure. Similar to cardiac output measurements, where a single number without context has little meaning, normalizing time spent on CPB to BSA provides patient context. The reasons for that are 3-fold: first, there is a greater haemodilution effect in smaller patients with a lower BSA. This fact, secondly, leads to a higher risk of red blood cell transfusion and associated complications in patients with lower BSA [21, 22]. Thirdly, prior work has proposed that an ‘obesity paradox’ may be present in cardiac surgery, where patients with a higher BMI (and therefore BSA) have better outcomes after cardiac surgery involving CPB than smaller patients [17]. In a cohort of 3560 patients undergoing CABG smaller patient size correlated with increased operative mortality, myocardial infarction, cerebrovascular accident and hospital LOS [23]. Several studies have demonstrated the association between increased time on CPB and poorer clinical outcomes. Analysis of a single-centre cohort of 5006 patients demonstrated an association between increased time on CPB and several patient-centred outcomes including mortality, renal dysfunction, neurological dysfunction and pulmonary dysfunction [13]. These cohort studies have been criticized for not taking into consideration that longer CPB times are often due to higher complexity, thus increasing morbidity and mortality associated with the operation.

Our study demonstrated the risk of increased morbidity with higher BI or longer CPB time, giving credence to the suggestion that expeditious surgery is not only associated with less morbidity but also better resource utilization.

We did not observe an association between bypass and mortality, which may be explained by our low mortality rate (0.6%). Salis *et al.* [13] observed 2.6% mortality in their cohort, the higher event rate allowed the association between duration of CPB and mortality. In view of the low event rate, we chose to use LOS as a surrogate marker for morbidity as our primary outcome rather than mortality. An analysis of administrative data from the Global Comparators Project, including over 4 million admissions, found that patients in the upper quartile of LOS had higher odds of mortality (OR: 1.45, 95% CI: 1.43–1.47) and a higher morbidity burden [24]. Postoperative LOS is patient-centred, institution-centred and population-centred. LOS may be influenced by confounding factors, however, at our institution discharge criteria are protocolized limiting the likelihood of this. To keep data as homogeneous as possible we restricted data analysis to operations before the first wave of SARS-CoV-2 started compromising healthcare in the UK, particularly hospital discharge of medically fit patients, which is still relevant to this day.

The BI of patients with a hospital stay ≥ median time in our cohort is significantly higher than that of patients with a shorter stay.

A multitude of risk factors for postoperative atrial fibrillation (AF) have been described over the years. Besides hypokalaemia [25] and hypomagnesaemia [26], no clear intraoperative risk factors have been identified. To the best of our knowledge, only one study investigated a possible association between duration of CPB and aortic cross-clamping and postoperative AF but yielded inconclusive results [27]. In our study, there was a significant association between above mean BI or CPB time and postoperative AF in all included patients and in the CABG-only group.

CPB-associated renal dysfunction accounts for the biggest burden of morbidity after cardiac surgery and is associated with a more than 2-fold increase in early mortality regardless of the definition used for acute kidney injury (AKI) [28]. The global incidence of AKI after cardiac surgery is 22.3% across all accepted definitions [29]. A single-centre review of 3575 patients in a German University Hospital showed that fewer patients develop AKI if surgery and ischaemic time is kept short, blood loss is kept to a minimum and CPB is conducted in normothermia [12]. A review of over 11 000 case records in Italy showed that time spent on CPB was associated with an increased risk in renal failure requiring renal replacement therapy. The statistical significance was lost after adjusting for confounders [30]. The results from our study corroborate earlier findings that longer CPB time is associated with an increased incidence of renal dysfunction.

In addition to ischaemia-reperfusion injury, the cause of postoperative pulmonary dysfunction is likely to be linked to anatomical and physiological factors Firstly, the lungs act as a filter in the venous circulation and therefore all active and activating substances generated during CPB will transit through them; secondly, the smaller lung capillaries are more prone to trapping debris and aggregates, which in turn leads to higher local activation of inflammatory mediators; thirdly, the lungs are home to a considerable pool of neutrophils. We did not see an association between BI and pulmonary dysfunction in our cohort, neither overall nor in the isolated CABG group. This might be explained by the fact that the triggers are an exposure effect rather than dose-dependent. Earlier studies exploring lung perfusion with protective solutions like Celsior during CPB and aortic cross-clamping have not led to improved postoperative lung function [31]. The fact that pulmonary function is equally not influenced by the inspired oxygen fraction of gas insufflated from the ventilator during aortic cross-clamping [32] might underpin the theory that ischaemia-reperfusion injury in addition to an overwhelming burden of inflammatory metabolites flooding the lung precipitate pulmonary dysfunction regardless of duration of CPB.

### Limitations

Our study has several limitations. First, we are not able to report on neurological outcomes such as postoperative delirium or confusion, which are known to increase ICU and postoperative LOS [33]. Since the group with a LOS above median stay has a significantly higher BI it is possible that neurological complications are a contributor to the increased LOS. We do not have the data for potential confounders, such as pre-existing dementia, but think that an additional study might be warranted.

Second, due to its retrospective nature, there is the possibility that missing or incorrect data may have influenced our results. Of 2413 eligible patients, only 119 were excluded due to incomplete data. CPB and patient demographic data are directly imputed on the day of surgery into the locally curated CARDS II database. Outcome variables were directly retrieved from the ICU electronic record-keeping system, and our outcome variables of interest were specifically selected to minimize the risk of data error. Despite this, it is conceivable that missing data may have impacted our results.

Third, this is a single-centre study and so may lack external validity. Our initial intention was to undertake a multicentre study. However, when other centres were approached, we encountered issues with data access, and they could not vouch for the completeness of their data sets. To preserve the integrity of our study, we decided to use a single, well-maintained dataset from a digitally mature institution so as not to negatively impact the validity of our findings.

Fourth, it is possible that the CPB time component of BI is confounded by other factors such as surgical complexity, or patient comorbidity. It remains plausible that unrecognized confounding factors may have influenced our results.

Fifth, we used a relatively short data collection period of 23 months. Data in the two large cohort studies was collected over 6 years, but it is possible that cardiac surgical, anaesthesia and CPB practice and equipment may have changed over this period, thereby possibly confounding results. Using a shorter inclusion period will have limited this effect.

Despite the above limitations, the study shows that reducing bypass time is likely to be associated with reduced LOS, better outcomes and less morbidity. It can, of course, be argued that long bypass times are dictated by the technical difficulty of a particular procedure, so that this factor alone is responsible for the adverse outcomes. This is true to some extent, but bypass times are also affected by the speed of individual surgeons, the choice of surgical technique and the addition of procedures the indications for which are borderline. In surgery generally, and cardiac surgery especially, speed is good provided no corners are cut and the final technical result is not compromised. Our study should raise awareness that unnecessary delay in the completion of the surgical procedure may have an adverse effect on outcomes and should be avoided where possible. When the delay is due to the patient, little can be done. When it is due to the surgeon, there may be room for improvement.

## CONCLUSION

In our cohort of 2294 patients undergoing cardiac surgery requiring CPB at an academic, quaternary referral cardiothoracic hospital, we tested a novel index of bypass exposure, the BI. Although we demonstrated that indexing CPB time to BSA was associated with postoperative LOS, ICU LOS, new renal injury and new cardiac rhythm disturbance, it did not appear to have a stronger association with these outcome measures than bypass time alone.

## Supplementary Material

ivae171_Supplementary_Data

## Data Availability

The data supporting this study’s findings are available from the corresponding author upon reasonable request. All data are held on a secure repository within the hospital network and can be made available in anonymized form.

## ABBREVIATIONS

AKI: Acute kidney injury
BI: Bypass index
BMI: Body mass index
BSA: Body surface area
CPB: Cardiopulmonary bypass
GAM: Generalized additive model
ICU: Intensive care unit
IQR: Interquartile range
LOS: Length of stay
SD: Standard deviation
