# Supplementary material for: Predictive role of cardiopulmonary bypass exposure indexed to body surface area on postoperative organ dysfunction: a retrospective cohort study
Source: Interdiscip Cardiovasc Thorac Surg. 2024 Oct 7;39(4):ivae171. doi: 10.1093/icvts/ivae171 (PMC11483134; doi:10.1093/icvts/ivae171)
Supplement: ivae171_Supplementary_Data [file ivae171_supplementary_data.docx]

**Supplemental material**

**PERFUSION TECHNIQUE**

CPB was conducted in accordance with standard institutional practice, using S5 Heart-Lung Machines with Sorin Inspire 8F oxygenator and phosphorylcholine coated polyvinylchloride tubing (LivaNova, London, UK). The priming fluid was 2 L Hartmann’s solution, 500 mL of 10% Mannitol and 5000 IU heparin. Once the circuit was primed and deaired, the priming volume was reduced by 1 L to avoid unnecessary hemodilution and to allow retrograde autologous priming technique (RAP) where appropriate. Operations were carried out in normothermia or mild hypothermia (37–32°C). CPB flow was maintained at 2.4 L/min/m^2^, hematocrit was kept >22% and vasopressors were used as required to maintain blood pressure between 60–80mmHg. Blood cardioplegia with St Thomas’ solution was used in all patients, with a 4:1 blood / cardioplegia ratio.

**ANAESTHESIA TECHNIQUE**

Sedative pre-medication was not used. Anesthesia was induced with a combination of benzodiazepine / opioid / propofol and maintained with a propofol infusion and additional inhaled anesthetics at the discretion of the consultant anesthesiologist in charge. Muscle relaxation was achieved using rocuronium (0.6-1 mg/kg) or pancuronium (0.1 mg/kg). A 2 g bolus of tranexamic acid was given intravenously either on induction or with heparin. After tracheal intubation all patients were ventilated with intermittent positive pressure with tidal volumes of 6 - 8ml/kg. During CPB, ventilation was stopped and the lungs were insufflated with O_2_/Air and no continuous positive airway pressure was applied. Where appropriate, the blood glucose level was kept 10mmol/l with a continuous insulin infusion. Antibiotic prophylaxis was administered during induction and following separation from CPB as per institutional protocol.

Before aortic cannulation a 300 IU/kg bolus of heparin was given to achieve safe anticoagulation and the activated clotting time (ACT) was maintained > 400 seconds throughout the duration of CPB using intermittent further heparin bolus doses as required.

In order to facilitate safe weaning from CPB, epicardial pacing, inotropes (dopamine, adrenaline), vasoconstrictors (noradrenaline, vasopressin), vasodilators (nitroglycerin) or inodilators (enoximone) were used as directed by the attending anesthesiologist and surgeon. Heparin was reversed with protamine (0.8 - 1mg protamine / 100IU heparin) once patient circulation had successfully been established. Following conclusion of surgery patients were transferred to ICU, where they were treated according to a nurse-led treatment algorithm for immediate postoperative patients. Once the hemodynamic, temperature and metabolic targets were achieved and there was no excessive chest tube drainage, sedation was stopped aiming for tracheal extubation at the earliest possible time.

| Table S1: Baseline variables and postoperative variables comparing patients with hospital length of stay < median to those with hospital length of stay ≥ median | | | |
| --- | --- | --- | --- |
|  | < Median LOS | ≥ Median LOS | P value |
| Male, n (%) | 872 (76.8%) | 797 (70.3%) | <0.001 |
| Female, n (%) | 264 (23.2%) | 336 (29.7%) | <0.001 |
| Age, mean (SD) | 67.2 (10.6) | 70.6 (11.1) | <0.001 |
| Height (cm), mean (SD) | 170.5 (9.2) | 169.3 (9.8) | 0.004 |
| Weight (kg), mean (SD) | 83.1 (16.3) | 82.2 (17.4) | 0.199 |
| BSA, mean (SD) | 1.98 (0.22) | 1.96 (0.24) | 0.059 |
| EuroSCORE additive, mean (SD) | 4.9 (2.6) | 6.6 (2.9) | <0.001 |
| EuroSCORE logistic, mean (SD) | 5.3 (5.9) | 9.0 (9.3) | <0.001 |
| EuroSCORE2, mean (SD) | 2.2 (3.2) | 4.1 (5.1) | <0.001 |
| Preop rhythm:  -Sinus, n (%)  -AF/Flutter, n (%)  -Other, n (%) | 998/1127 (88.6%)  106/1127 (9.4%)  23/1127 (2.0%) | 874/1118 (78.2%)  207/1118 (18.5%)  37/1118 (3.3%) | <0.001 |
| Total bypass time (min), mean (SD) | 99.6 (40.2) | 117.2 (69.4) | <0.001 |
| Bypass index, mean (SD) | 50.9 (20.9) | 60.6 (38.1) | <0.001 |
| Any organ dysfunction, n (%) | 388/1136 (34.2%) | 555/1133 (49.0%) | <0.001 |
| New postop rhythm dysfunction, n (%) | 66/1136 (5.8%) | 233/1133 (20.6%) | <0.001 |
| Postop renal dysfunction, n (%) | 30/1136 (2.6%) | 56/1133 (5.0%) | 0.003 |
| Postop CRRT, n (%) | 10/1136 (0.9%) | 21/1133 (1.9%) | 0.046 |
| Postop pulmonary dysfunction, n (%) | 327/1136 (28.8%) | 386/1133 (34.1%) | 0.007 |
